# The Stroma Liquid Biopsy Panel Contains a Stromal-Epithelial Gene Signature Ratio That Is Associated with the Histologic Tumor-Stroma Ratio and Predicts Survival in Colon Cancer

**DOI:** 10.3390/cancers14010163

**Published:** 2021-12-29

**Authors:** Cor J. Ravensbergen, Matthew Kuruc, Meaghan Polack, Stijn Crobach, Hein Putter, Hans Gelderblom, Devjit Roy, Rob A. E. M. Tollenaar, Wilma E. Mesker

**Affiliations:** 1 Department of Surgery, Leiden University Medical Center, Albinusdreef 2, 2333 ZA Leiden, The Netherlands; c.j.ravensbergen@lumc.nl (C.J.R.); M.Polack@lumc.nl (M.P.); r.a.e.m.tollenaar@lumc.nl (R.A.E.M.T.); 2 Biotech Support Group LLC, 1 Deer Park Drive, Suite M, Monmouth Junction, NJ 08852, USA; mkuruc@biotechsupportgroup.com; 3 Department of Pathology, Leiden University Medical Center, Albinusdreef 2, 2333 ZA Leiden, The Netherlands; A.S.L.P.Crobach@lumc.nl; 4 Department of Medical Statistics, Leiden University Medical Center, Albinusdreef 2, 2333 ZA Leiden, The Netherlands; H.Putter@lumc.nl; 5 Department of Medical Oncology, Leiden University Medical Center, Albinusdreef 2, 2333 ZA Leiden, The Netherlands; a.j.gelderblom@lumc.nl; 6 Hospital Medicine, Nathan Littauer Hospital, 99 East State Street, Gloversville, NY 12078, USA; devjit.roy@gmail.com

**Keywords:** colon cancer, tumor microenvironment, tumor-stroma ratio, gene signature, liquid biopsy, Stroma Liquid Biopsy^TM^, biomarker

## Abstract

**Simple Summary:**

Liquid biopsy offers a novel minimally invasive approach to tumor sampling and is believed to capture a comprehensive overview of the molecular tumor landscape. However, current liquid biopsy analytes in cancer are principally derived from the malignant cells without regard to the tumor microenvironment. The Stroma Liquid Biopsy^TM^ (SLB) proteomics panel contains proteins from key stromal pathways in cancer and was designed to address the tumor microenvironment in liquid biopsy. We aimed to explore and characterize SLB panel constituents using an in-silico transcriptomics approach in colon cancer. Additionally, the association between the SLB panel constituents and histologic intratumoral stromal content, a poor prognostic tumor characteristic, was investigated. This explorative study presents an alternative workflow to gene signature development and provides a molecular characterization of the SLB panel. We believe that our findings contribute to the ever-increasing appreciation of the tumor microenvironment in cancer.

**Abstract:**

Liquid biopsy has emerged as a novel approach to tumor characterization, offering advantages in sample accessibility and tissue heterogeneity. However, as mutational analysis predominates, the tumor microenvironment has largely remained unacknowledged in liquid biopsy research. The current work provides an explorative transcriptomic characterization of the Stroma Liquid Biopsy^TM^ (SLB) proteomics panel in colon carcinoma by integrating single-cell and bulk transcriptomics data from publicly available repositories. Expression of SLB genes was significantly enriched in tumors with high histologic stromal content in comparison to tumors with low stromal content (median enrichment score 0.308 vs. 0.222, *p* = 0.036). In addition, we identified stromal-specific and epithelial-specific expression of the SLB genes, that was subsequently integrated into a gene signature ratio. The stromal-epithelial signature ratio was found to have prognostic significance in a discovery cohort of 359 colon adenocarcinoma patients (OS HR 2.581, 95%CI 1.567–4.251, *p* < 0.001) and a validation cohort of 229 patients (OS HR 2.590, 95%CI 1.659–4.043, *p* < 0.001). The framework described here provides transcriptomic evidence for the prognostic significance of the SLB panel constituents in colon carcinoma. Plasma protein levels of the SLB panel may reflect histologic intratumoral stromal content, a poor prognostic tumor characteristic, and hence provide valuable prognostic information in liquid biopsy.

## 1. Introduction

The tumor microenvironment (TME), or stroma, refers to the local environment in which cancer cells are embedded and comprises a multitude of (sub)cellular components [1]. The dynamic interactions that occur between the TME and the malignant cells promote tumorigenesis and cancer progression [2]. Recognition of these dynamic interactions, which encompass biological processes such as inflammation, neoangiogenesis, and extracellular matrix (ECM) degradation, has led to widespread scientific interest in utilizing the TME for clinical applications. Indeed, the histologic tumor-stroma ratio (TSR), a stroma-derived biomarker developed by our group, has been validated as an independent predictor of patient survival in various primary tumor types [3,4,5,6,7,8,9,10,11,12,13]. Moreover, the TSR was found to be associated with pathologic response to neoadjuvant therapy in esophageal and breast cancer, supporting the notion that the TME modulates therapeutic response [6,14,15].

Considering predictive biomarkers in cancer prognosis and treatment response, recent scientific efforts have focused on liquid biopsy as a novel tool in cancer diagnostics. Liquid biopsy refers to the sampling of analytes from non-solid tissue specimens. Being minimally invasive, liquid biopsy offers a major advantage in sample accessibility as opposed to traditional methods, such as solid tissue biopsy [16]. In addition, liquid biopsy is believed to capture a comprehensive overview of the tumor landscape, compensating for the loss of information concerning the vast intratumoral heterogeneity when performing solid tissue biopsy [17]. Significant advances in malignant cell-derived biomarkers, such as circulating tumor cells (CTCs) and circulating cell-free tumor DNA (ctDNA), have led to successful tumor profiling through liquid biopsy, which currently awaits clinical approval [18,19].

Despite these promising features, the TME has remained underappreciated in current liquid biopsy research. To address TME profiling in liquid biopsy, the *Stroma Liquid Biopsy*^TM^ (SLB) panel was developed as an experimental stroma-oriented proteomics alternative to the conventional genomic liquid biopsy biomarkers in oncology [20]. The SLB panel comprises a set of key proteins in interconnected stromal pathways (i.e., coagulation, acute phase inflammation) and is believed to capture a deranged systemic response to the presence of cancer in a plasma proteomic blueprint [20,21]. In the current explorative work, we performed a transcriptomic characterization of SLB panel constituents in publicly available datasets of colon carcinoma samples. The findings in this study provide new insights into the components of the SLB panel and emphasize the versatility and significance of TME factors in cancer biomarker research. 

## 2. Materials and Methods

### 2.1. Data Collection, Processing and Patient Cohort Selection

We retrospectively analyzed gene expression profiles obtained from The Cancer Genome Atlas (TCGA) and Gene Expression Omnibus (GEO). Illumina HiSeqV2 Level 3 mRNA bulk sequencing data and curated clinical metadata from the Pan-Cancer Atlas COAD project were obtained from the Genomic Data Commons (GDC; https://gdc.cancer.gov, accessed on 1 April 2021) by R/Bioconductor package TCGAbiolinks (version 2.18.0) [22]. Only curated clinical endpoints, as described by Liu et al., were used in this study to assure high-quality analyses [23]. Microsatellite instability (MSI) scores for the TCGA Pan-Cancer Atlas COAD project were obtained from the original publication by Ding et al. [24]. The MSI score was determined using MSIsensor software (version 1.0) [25]. MSI was defined as an MSI score of ≥4 [24]. Patient identifiers of the included cohort are available in Supplementary Data S1. All patients were therapy-naive upon data acquisition. Absolute gene expression data was gene length normalized and expressed as log-transformed transcripts per million (TPM). 

For the validation cohort, level 3 normalized Affymetrix Human Genome U133 Plus 2.0 Array data and clinical metadata from the Smith cohort [26] (accession: GSE17538) were downloaded from GEO (https://www.ncbi.nlm.nih.gov/geo/, accessed on 1 April 2021). Affymetrix probes were mapped to Human Genome Organization (HUGO) gene symbol, when multiple probes were mapped to the same gene symbol, the highest signal value was used to represent its expression level. Signal values for all probes were log-transformed. Baseline characteristics and population composition of the TCGA and GEO cohorts can be found in Supplementary Table S1. 

### 2.2. Gene Selection, Functional Enrichment and Protein-Protein Interaction Network Analysis

Based on the proteins of the SLB panel, we selected the corresponding genes for inclusion in this study (Table 1). Gene Ontology (GO) enrichment analysis was performed on the selected genes [27,28,29]. Significantly enriched biological process (BP) categories were defined as having a false discovery rate (FDR) of <0.05. A protein-protein interaction (PPI) network was constructed to identify hub genes/proteins and to assess functional relations. The initial PPI was constructed with the Search Tool for the Retrieval of Interacting Genes (STRING) database (http://www.string-db.org/, accessed on 10 April 2021) and then exported to Cytoscape software (version 3.8.2) for further network and K-means 3-cluster analyses using the embedded Analyze Network function. 

### 2.3. Gene Set Enrichment Analysis and Consensus Molecular Subtypes

Single sample gene set enrichment analysis (ssGSEA) was performed on the normalized gene expression data to define enrichment of the SLB gene set and specific stromal and immune pathways related to the SLB panel [30]. The analyses were performed using the open-source GSVA package for R [31]. The stromal and immune signaling pathway gene sets used in this study were obtained from the hallmark gene set collection of the Molecular Signatures Database (MSigDB) [32]. All gene sets used in this study are available in Supplementary Data S1. The consensus molecular subtypes (CMS) of colorectal cancer were computed on the normalized gene expression matrix using the CMSclassifier R package [33]. The single sample predictor was used, and CMS labels were assigned based on the nearest CMS output. Subsequently, the novel computed CMS labels were verified in the pre-computed CMS label dataset provided by the original authors [33]. CMS labels are available in Supplementary Data S1. 

### 2.4. Gene Signature Development and Prognostic Risk Model Establishment

To assess the expression of the stromal gene set on a single-cell level, we analyzed a publicly available single-cell RNA (scRNA) sequencing dataset from a comprehensive analysis of TME cell populations in colorectal cancer (CRC) patients [34]. The dataset contained gene expression profiles from >5000 cells acquired from 10 therapy-naive stage II/III CRC patients and was accessed via an interactive web tool (http://crcleukocyte.cancer-pku.cn/, accessed on 1 May 2021). The dataset contained 8 major cell clusters that were identified based on canonical cell markers as described by Zhang et al. [34]. The geometric mean of the expression of the total stromal gene set was computed per cell and visualized as a boxplot to demonstrate cell population involvement. Relative expression of genes per cell population was visualized in a heatmap using Z-scored mean expression values standardized per gene (Supplementary Data S2). Subsequently, the inclusion of genes into a signature was based on a Z-score of >1.5. 

Following the identification of stromal and epithelial signatures, a patient-specific risk score was calculated, for the signatures separately, as the sum of the absolute gene expression level of each gene, multiplied by the corresponding regression coefficient derived from Cox multiple regression analysis of the OS. The separate risk scores were then combined into a ratio-risk score according to the equation found in Supplementary Figure S1. To correct for variations in absolute gene expression levels due to different mRNA sequencing methods, the regression coefficients were computed per cohort (Supplementary Table S2). Patients were stratified in high- and low ratio-risk scores based on the median score as the cut-off value. The cohort computed regression coefficients and cut-off values were then applied to all subpopulation analyses. Stromal-, epithelial- and ratio-risk scores of the TCGA COAD and GSE17538 cohorts are available in Supplementary Data S1. 

### 2.5. Tumor-Stroma Ratio

For TSR scoring, diagnostic Hematoxylin and Eosin (H&E)-stained slides of primary tumors from the TCGA COAD project were downloaded through the GDC portal. Using Aperio Imagescope (version 12.4.3) digital slide viewer software, the visual field with the highest amount of stroma was selected, according to the previously published protocol [35]. The percentage of stroma was scored in increments per ten percent and the tumors were subsequently categorized as stroma-high (>50% stroma) or stroma-low (≤50% stroma) using the standardized cut-off value of 50% [3]. To ensure proper TSR scoring, the observers (C.R. and M.P.) were trained with the TSR E-learning module constructed for the Uniform Noting for International Application of the Tumor-Stroma Ratio as an Easy Diagnostic Tool (UNITED) study [36,37]. In 33% percent of the slides, blinded visual scoring was performed by a second observer (M.P.) and subsequently, the interobserver agreement was assessed by Cohen’s kappa coefficient. If consensus could not be reached, scoring by a third observer (S.C., board-certified pathologist) was decisive. The scored stromal percentages are publicly available in Supplementary Data S1.

### 2.6. Statistical Analysis

The R programming language (version 4.0.5; https://www.r-project.org/) was used for statistical analysis and data visualization (packages tidyverse, viridis, survival and pROC). Variable distribution was assessed with the Shapiro-Wilk test, followed by either parametric or non-parametric testing. To detect statistically significant differences in baseline characteristics between cohorts, Fisher’s exact test or the chi-squared test were used for categorical variables and the Mann–Whitney U test was used for continuous variables. The log-rank test was used to compare survival distributions and Kaplan–Meier survival curves were plotted with a 95% confidence interval. Cox regression was performed for multivariate analysis. Hazard ratios (HR) were calculated with a 95% confidence interval. Interobserver variability for TSR scoring was evaluated with Cohen’s Kappa coefficient. A two-tailed *p*-value of ≤0.05 was considered statistically significant.

## 3. Results

### 3.1. Sample Selection and Patient Characteristics

**Figure 1:**
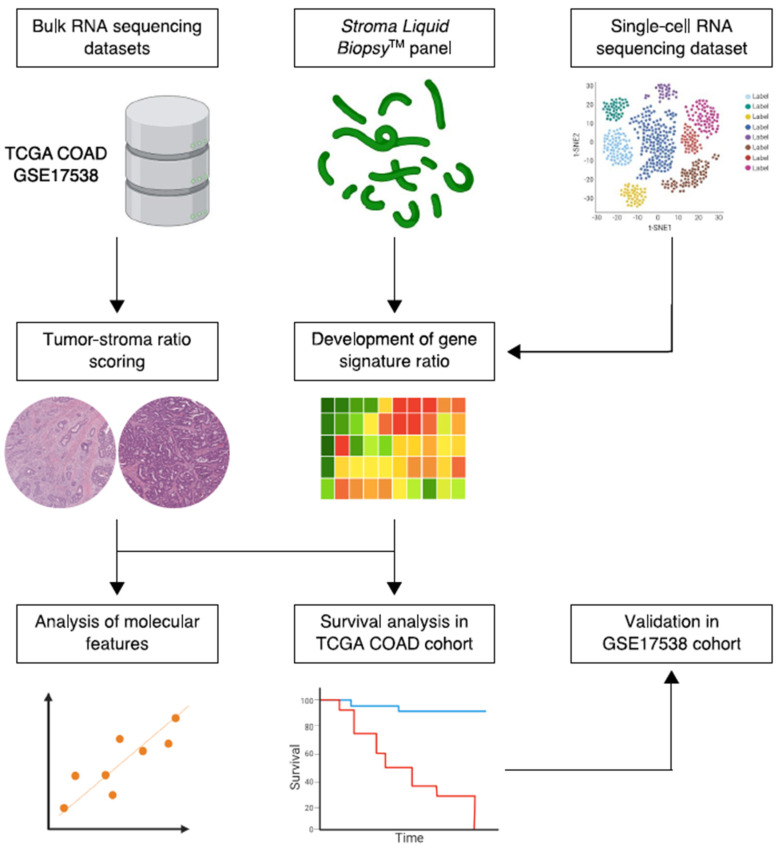
Simplified schematic workflow of the study. Bulk and single-cell RNA sequencing data were collected from public repositories. Constituents of the Stroma Liquid Biopsy panel were used to develop a stromal-epithelial gene signature ratio based on single-cell gene expression of intratumoral cell populations. The histologic tumor-stroma ratio was used to quantify intratumoral stromal content in pathology slides from the TCGA COAD cohort. Subsequently, the gene signature ratio was studied in histologic stroma-high and stroma-low tumors. In addition, the association between the gene signature ratio and established molecular tumor characteristics, such as microsatellite instability and the consensus molecular subtypes, was assessed. Lastly, the prognostic performance of the gene signature ratio was tested in the TCGA COAD discovery cohort and validated in the GSE17358 cohort.

A total of 359 samples of the Pan-Cancer Atlas COAD project from TCGA were selected for the discovery set. Inclusion was based on colon adenocarcinoma histological subtype, complete pathologic stage, and complete overall survival (OS) follow-up metadata. In addition, patients were required to be therapy-naïve upon tumor sampling. An external validation cohort was selected from the GEO GSE17538 dataset, consisting of 229 adenocarcinoma samples with complete pathologic stage and OS follow-up data as well, previously reported by Smith et al. [26]. The test and validation cohorts demonstrated a similar distribution for TNM-staging. In addition, all patients in the current study were therapy-naive at the time of tissue sampling. Additional patient characteristics of the respective datasets can be found in Supplementary Table S1. A simplified schematic workflow of our study is shown in Figure 1.

### 3.2. Gene Set Enrichment Analysis Demonstrates Increased Expression of SLB Panel Genes in Histologic Stroma-High Tumors

A total of 13 stroma-derived genes from the SLB panel were included for analysis (Table 1). Based on literature study, the selected genes were shown to be involved in dysregulated stromal pathways in cancer (i.e., coagulation, acute-phase inflammation, and the complement cascade) [20]. GO analysis demonstrated significant enrichment of the gene set in TME-associated biological processes, namely complement activation, platelet degranulation, leukocyte migration, and angiogenesis (Supplementary Table S3). A PPI network was constructed to assess the stromal gene (product) interactions (Supplementary Figure S2A). Node degree revealed C3, TIMP1 and SERPINA1 as hub genes in the constructed network. ECM1, CHGA and SAA2 shared no functional relations with the network (Supplementary Table S4). Subsequent clustering analysis of the network revealed three distinct functional clusters, consistent with the literature functional annotation as described above (Supplementary Figure S2B).

**Figure 2:**
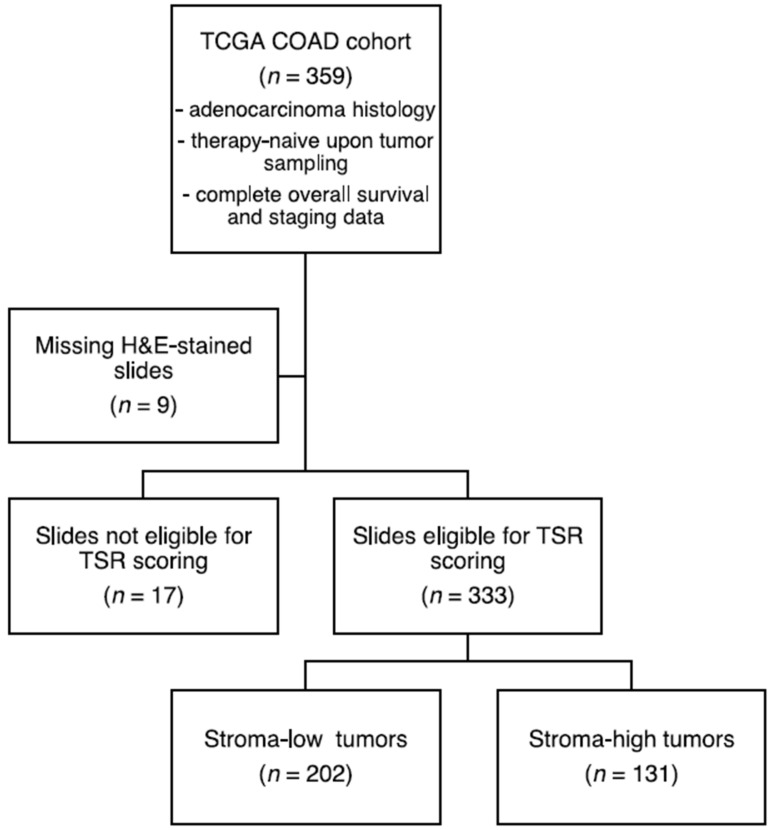
Flowchart of histologic tumor-stroma ratio (TSR) scoring. H&E, hematoxylin & eosin.

**Figure 3:**
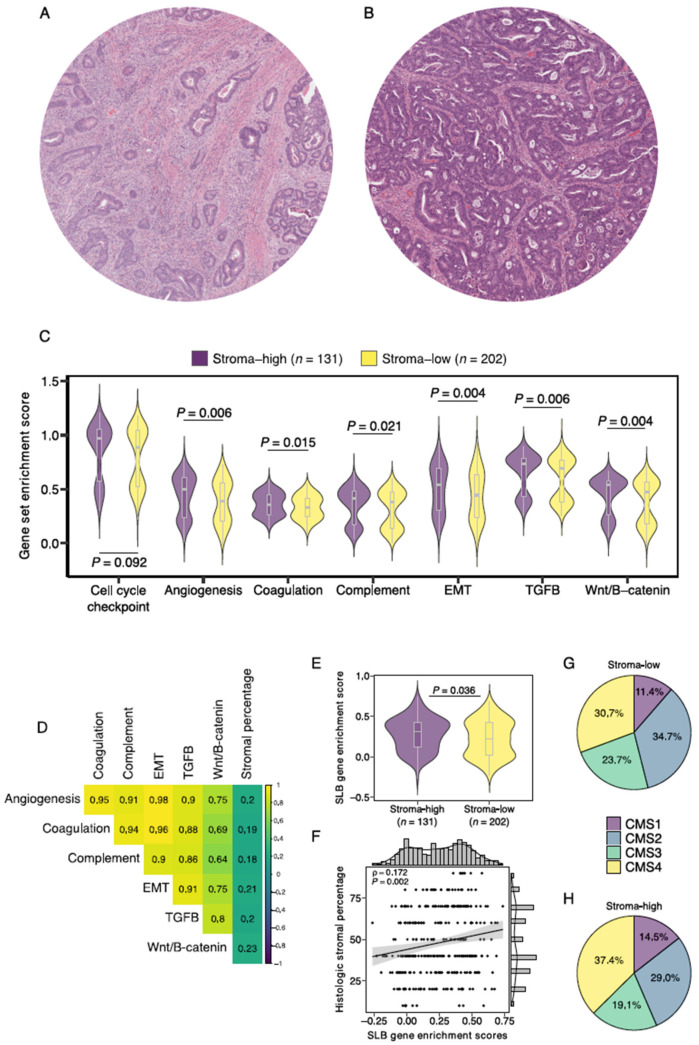
Gene enrichment analysis of stromal pathways and its association to histologically scored stromal percentage. Illustrative images of (**A**) stroma-high and (**B**) stroma-low tumors as scored by the TSR. (**C**) Gene enrichment scores of 6 notable stromal pathways for stroma-high and stroma-low tumors. (**D**) Correlation matrix of stromal pathway gene enrichment scores and histologic stromal percentage. (**E**) Enrichment score of the Stromal Liquid Biopsy gene set in stroma-high and stroma-low tumors. (**F**) Correlation plot of Stromal Liquid Biopsy gene enrichment score and histologic stromal percentage. (**G**) Distribution of consensus molecular subtype (CMS) in histologic stroma-low tumors. (**H**) Distribution of CMS in histologic stroma-high tumors. EMT, epithelial-to-mesenchymal transition; SLB, Stromal Liquid Biopsy; ρ, Spearman’s rho.

We then evaluated the relationship between the SLB panel genes and the histologic TSR. A total of 333 (93%) slides proved to be of sufficient quality for TSR assessment (Figure 2). The Cohen’s kappa coefficient for interobserver reliability was 0.85, indicating strong agreement [38]. A total of 12 (4%) slides required a third review by an independent observer to reach a complete agreement. Baseline characteristics of the stroma-high and stroma-low populations can be found in Supplementary Table S5. We observed stroma-high and stroma-low tumors in 39.3% (131) and 60.7% (202) of the cases, respectively. This distribution is comparable to that of previous TSR studies in colon carcinoma [39]. Representative illustrations of stroma-high and stroma-low tumors can be found in Figure 3A,B.

First, we performed ssGSEA of 6 curated gene sets of biologically well-defined processes involved in stromal pathways to study the relationship between histologic stromal content and stroma-related gene expression. As a quality control step, a housekeeping cell cycle checkpoint gene pathway demonstrated no significant differences between stroma-high and stroma-low tumors (*p* = 0.092; Figure 3C). We found increased enrichment scores of all six stroma-related gene sets in stroma-high versus stroma-low tumors, angiogenesis (0.392 vs. 0.496, *p* = 0.006), coagulation (0.328 vs. 0.353, *p* = 0.015), complement system (0.379 vs. 0.416, *p* = 0.021), epithelial-to-mesenchymal transition (0.445 vs. 0.544, *p* = 0.004), TGF-*ß* signaling (0.691 vs. 0.737, *p* = 0.006), and Wnt/*ß*-catenin signaling (0.476 vs. 0.540, *p* = 0.004; Figure 3C). In addition, enrichment scores of all 6 gene sets demonstrated weak positive correlations with the consensus observer-scored histologic stromal percentages (Figure 3D). We then studied enrichment scores of the SLB panel gene set and found a significantly higher median score in the stroma-high tumors compared to the stroma-low tumors (median 0.308 vs. 0.222, *p* = 0.036; Figure 3E). SLB panel enrichment scores were weak positively correlated with continuous histologic stromal percentage (Rho = 0.172, *p* = 0.001; Figure 3F). These results indicate that stroma-related transcriptomic pathways demonstrate increased activity in tumors with histologic high stromal content.

The CMS classification of CRC describes a gene expression-based classification system that allows the categorization of tumors into one of four robust molecular subtypes [33]. The association between the CMS and histologic intratumoral stromal content was studied. Stroma-high tumors demonstrated a slightly larger proportion of CMS1 and CMS4 labels and a smaller proportion of CMS2 and CMS3 labels than stroma-low tumors (Figure 3G–H). Nevertheless, the distribution of CMS tumors in stroma-high and stroma-low tumors was not statistically significantly different, suggesting that the two categorization systems likely capture different populations at risk (*X*^2^ test = 15.73, *p* = 0.073).

### 3.3. Identification of a Gene Signature Ratio Based on Single-Cell Transcriptomics Data

**Figure 4:**
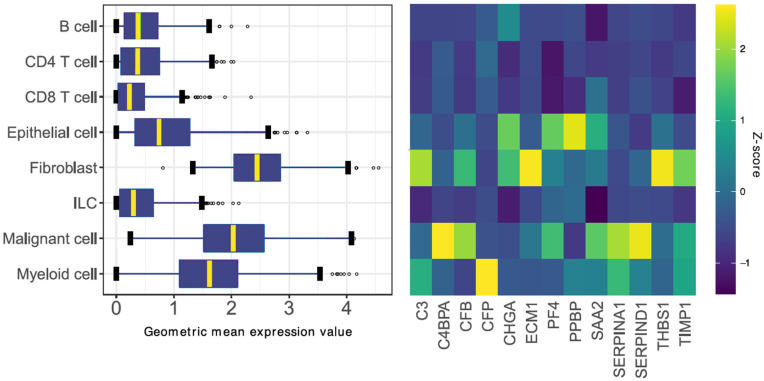
Identification of the stromal and epithelial signatures. (Left Panel) Boxplot summary of 8 major cell populations expressing the 13 stroma-derived genes in colorectal tumors. The boxplots visualize the geometric mean expression value of the pooled 13 genes per cell. (Right Panel) Heatmap of relative gene expression standardized per gene. Genes associated with high (relative) expression in stromal-phenotypic cells (i.e., fibroblasts and myeloid cells) were included in the stromal signature. Genes associated with high expression in epithelial-phenotypic cells (i.e., epithelial and malignant cells) were included in the epithelial signature. ILC, innate lymphoid cell.

To account for intratumoral cellular heterogeneity, we aimed to evaluate the expression of the SLB panel gene set in distinct cell populations within the tumor. The expression of the SLB genes was studied in a scRNA sequencing dataset of CRC tumors, previously reported by Zhang et al. [34]. We noticed a higher expression of the gene set in cells of epithelial- (i.e., colon epithelial and malignant cells), mesenchymal- (i.e., fibroblast), and myeloid-origin, as compared to lymphoid-derived cell types (Figure 4, left panel). Upon closer inspection, gene expression patterns related to epithelial-phenotypic cells and stroma-related cells were detected (Figure 4, right panel). Subsequently, we created two gene signatures using the expression Z-score of >1.5 as a cut-off for gene inclusion into the signatures (Supplementary Data S2); the stromal signature was composed of genes with relatively high expression in fibroblasts and myeloid cell types (C3, CFP, ECM1, THBS1, and TIMP1), whereas the epithelial signature was composed of genes with high expression in epithelial and malignant cell types (C4BPA, CFB, CHGA, PF4, PPBP, SAA2, SERPINA1, and SERPIND1).

### 3.4. TIMP1, PF4 and SERPINA1 Are Associated with Patient Survival and Share a Role in Platelet Degranulation

**Figure 5:**
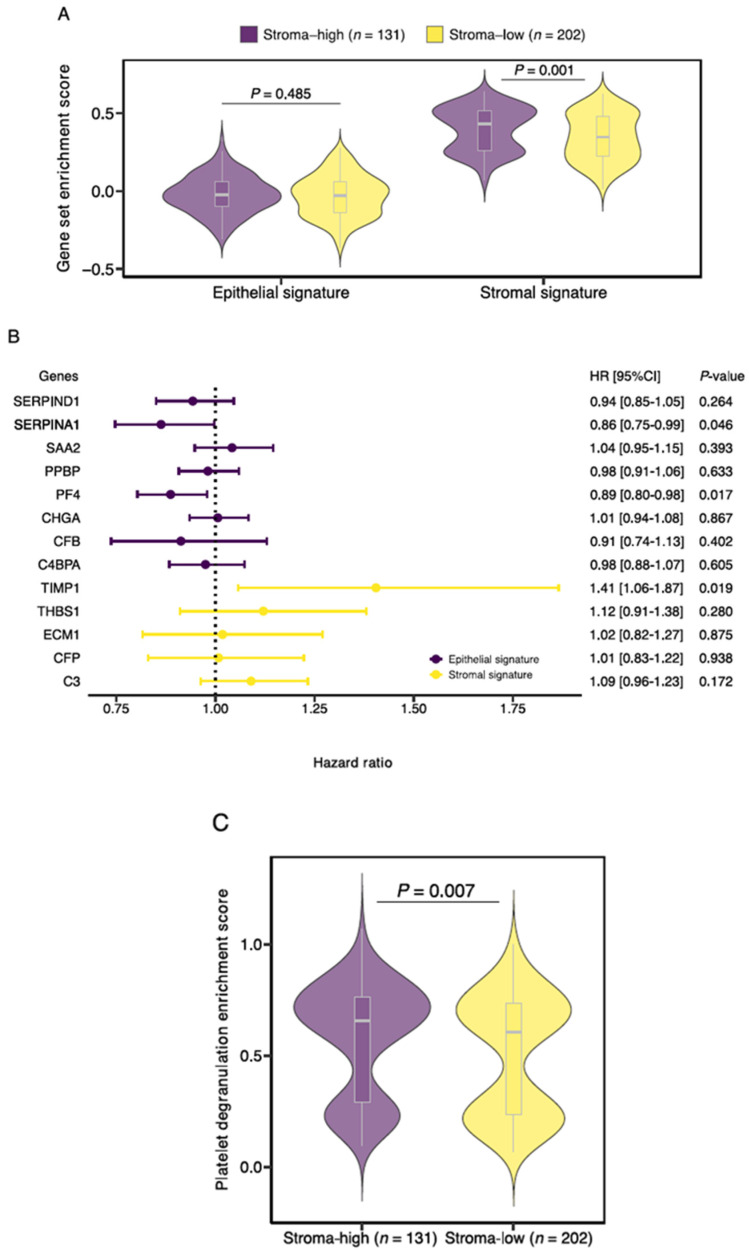
Characterization of the Stromal Liquid Biopsy (SLB) panel genes. (**A**) Gene enrichment scores of the newly defined stromal and epithelial signatures for stroma-high and stroma-low tumors. (**B**) Forest plot of univariate Cox regression analyses for the overall survival of the 13 SLB genes included in this study. (**C**) Platelet degranulation pathway enrichment score for stroma-high and stroma-low tumors. HR, hazard ratio.

Subsequently, the expression of the stromal and epithelial signatures was studied in histologic stroma-high and stroma-low tumors. Although gene enrichment scores for the epithelial signature were not significantly different between stroma-high and stroma-low tumors (median −0.024 vs. −0.029, *p* = 0.485), enrichment scores for the stromal signature were higher in the stroma-high tumors in comparison to the stroma-low tumors (median 0.432 vs. 0.346, *p* = 0.001; Figure 5A).

Next, we tested the contribution of the individual genes on patient OS (Figure 5B). A total of 3 of the 13 genes demonstrated a significant effect on survival. Expression of TIMP1, a constituent of the stromal signature, was associated with an increased risk of death (HR 1.405, 95%CI 1.058–1.866, *p =* 0.019). Conversely, expression of PF4 (HR 0.887, 95%CI 0.803–0.979, *p* = 0.017) and SERPINA1 (HR 0.863, 95%CI 0.747–0.997, *p* = 0.046), genes from the epithelial signature, was associated with decreased risk of death. Based on the earlier conducted functional enrichment analysis, we noticed that TIMP1, PF4 and SERPINA1 share a role in platelet degranulation, suggesting a key role for this biological process in colon cancer survival. Interestingly, when we performed gene set enrichment analysis of the curated platelet degranulation gene set, we observed increased enrichment scores in the histologic stroma-high tumors in comparison to the stroma-low tumors (median 0.654 vs. 0.605, *p* = 0.007; Figure 5C).

### 3.5. Establishment of the Prognostic Risk Model Reveals the Stromal-Epithelial Signature Ratio as a Predictor of Survival

Noticing the resemblance to the histologic TSR, we set out to establish a prognostic risk model utilizing the ratio of the developed stromal and epithelial gene signatures, the stromal-epithelial signature ratio. Based on a commonly applied gene expression-weighted risk score equation, a ratio-risk score was calculated for each patient. The median ratio-risk score was used as a cut-off, separating the cohort into high and low ratio-risk score groups (Supplementary Figure S3A). No significant differences were observed in the median ratio-risk score between different TNM stages (Supplementary Figure S3B).

**Figure 6:**
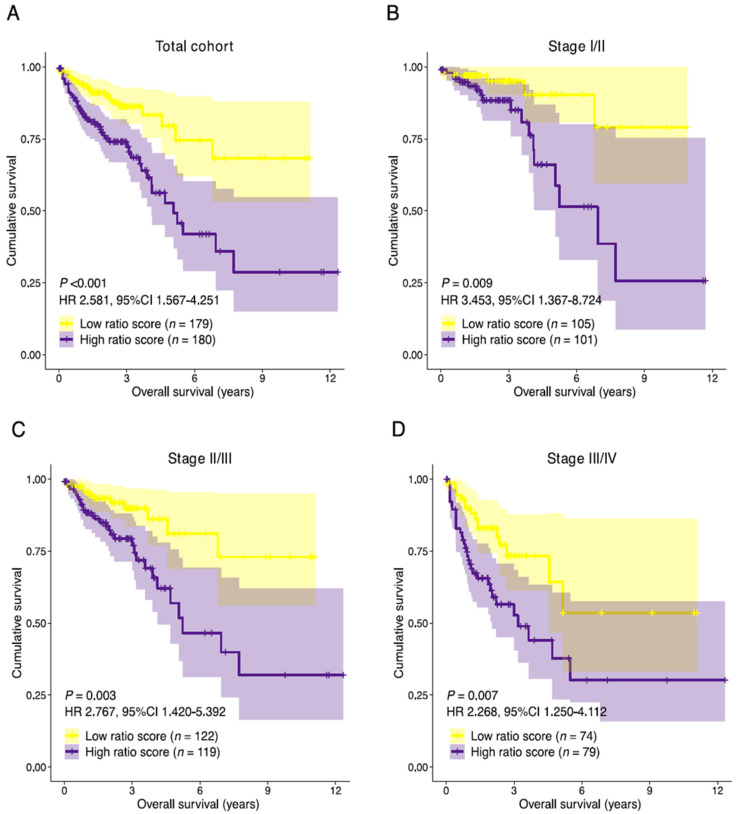
Prognostic performance of the stromal-epithelial signature ratio. Kaplan-Meier curves of the overall survival for the stromal-epithelial ratio-risk scores in (**A**) the total cohort (*n* = 359), (**B**) stage I/II (*n* = 206), (**C**) stage II/III (*n* = 241), and (**D**) stage III/IV (*n* = 153) subpopulations. Patients with a high ratio-risk score, categorized by the 50th percentile (median) cut-off, demonstrated a shorter overall survival time. HR, hazard ratio; CI, confidence interval.

The resulting ratio-risk score stratification was then tested for prognostic performance in the total discovery cohort and selected subpopulations. The median follow-up time of the 359 patients was 22.3 months. Patients with a high ratio-risk score had a lower median OS than patients with a low ratio-risk score (21.2 and 24.3 months, respectively). A high ratio-risk score was associated with statistically significantly shorter OS in the total cohort (HR 2.581, 95%CI 1.567–4.251, *p* < 0.001) and stage I/II combined (HR 3.453, 95%CI 1.367–8.724, *p =* 0.009), stage II/III (HR 2.767, 95%CI 1.420–5.392, *p =* 0.003) and stage III/IV (HR 2.268, 95%CI 1.250–4.112, *p =* 0.007) combined subpopulations (Figure 6A–D). The area under the receiver operating characteristic curve (AUC) for the prediction of the overall survival was 0.659 (total cohort), 0.682 (stage I/II), 0.672 (stage II/III), and 0.635 (stage III/IV) indicating a slight loss of performance in late-stage disease (Supplementary Figure S4A–D). Univariate analyses showed statistically significant associations between survival and the covariates age, pathological TNM stage, pathological T-stage, pathological N-stage and the stromal-epithelial signature ratio (Supplementary Table S6). After adjusting for clinical covariates, the stromal-epithelial signature ratio remained an independent predictor of OS (HR 2.586, 95%CI 1.561–4.286, *p* < 0.001). Unfortunately, due to poor data curation of treatment parameters in the TCGA COAD dataset, we were not able to correct for treatment type. Although no definitive assumptions can be made, the baseline characteristics between the high and low ratio-risk score groups did not significantly differ (Supplementary Table S7). 

Since 3 out of the 13 genes comprising the signature ratio demonstrated an individual significant effect on patient OS, we hypothesized that the prognostic performance of the signature ratio could potentially be improved by excluding the non-significant genes. Strikingly, we observed a minor decrease in prognostic performance when only TIMP1, PF4 and SERPINA1 were retained in the signature ratio (HR 1.769, 95%CI 1.102–2.841, *p* = 0.018; AUC = 0.639).

In addition to OS, the prognostic performance of the ratio-risk scores on the disease-specific survival (DSS) was analyzed. DSS data were not available for 8 patients, leaving a total of 351 patients available for DSS analysis. A high ratio-risk score was associated with significantly shorter DSS time in the total cohort (HR 2.099, 95%CI 1.169–3.770, *p =* 0.013; Supplementary Figure S5A,B). Due to the relatively short follow-up time in the TCGA COAD cohort and consequently a low number of events additional subpopulation analyses were not performed. Nevertheless, a high ratio-risk score remained an independent predictor of survival after adjusting for clinical covariates (HR 2.107, 95%CI 1.162–3.817, *p* = 0.014; Supplementary Table S8). 

### 3.6. Association with Established Histologic and Molecular Tumor Characteristics

**Figure 7:**
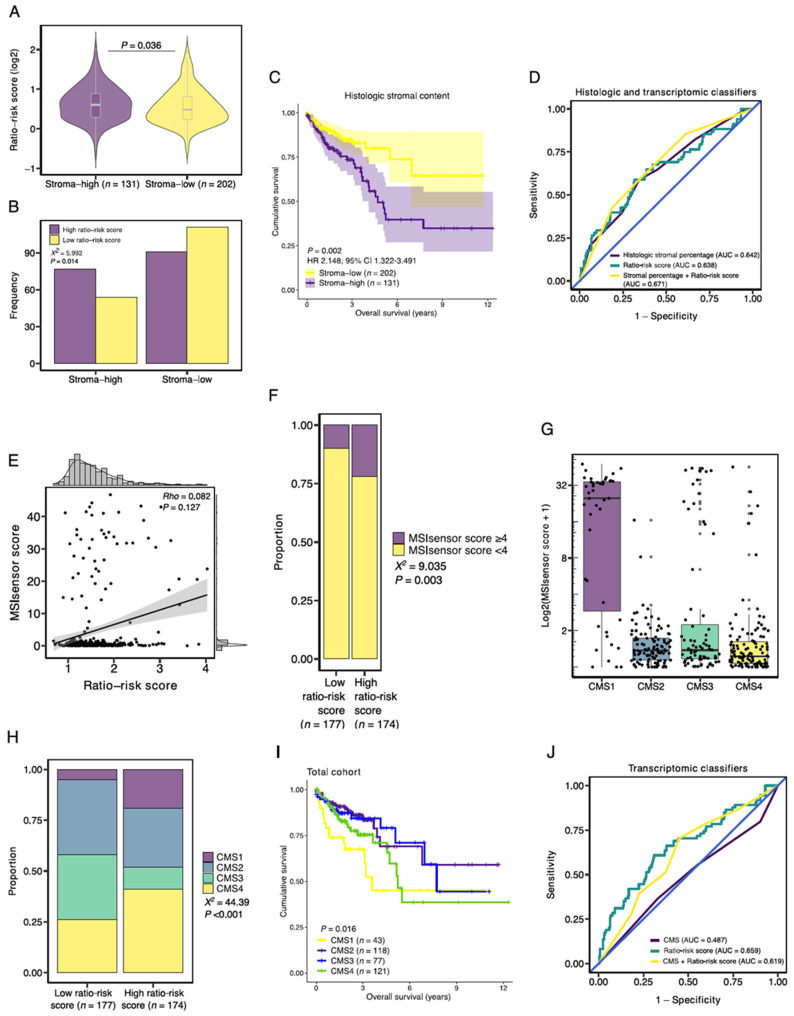
Association of the stromal-epithelial signature ratio with established histologic and molecular tumor characteristics. (**A**) Median ratio-risk score for the stroma-high and stroma-low populations as categorized by the TSR. (**B**) Frequency barplot for high and low ratio-risk scores stratified by the TSR. (**C**) Kaplan-Meier curve of the overall survival for the histologic stroma-high and stroma-low tumors. (**D**) Prognostic performance of histologic intratumoral stromal percentage and the ratio-risk scores. (**E**) Correlation between the continuous MSIsensor scores and ratio-risk scores. (**F**) Proportion of MSIsensor scores in the high and low-ratio risk score tumors. (**G**) MSIsensor scores and consensus molecular subtypes (CMS). (**H**) Proportion of consensus molecular subtypes (CMS) in the high and low-ratio risk score tumors. (**I**) Kaplan-Meier curve of the overall survival for the CMS. (**J**) Prognostic performance of the CMS and the ratio-risk scores. AUC, area under the curve; X2, Pearson Chi-Square; Rho, Spearman’s rho.

To assess the association between the signature ratio and histologic stromal content, we studied the ratio-risk scores stratified by stromal content and found an increased median ratio-risk score in stroma-high tumors in comparison to stroma-low tumors (1.52 vs. 1.39; *p* = 0.036; Figure 7A). In addition, stroma-high tumors more often had a high ratio-risk score (77 vs. 54), whereas a low ratio-risk score was more frequent in stroma-low tumors (111 vs. 91, *p* = 0.014; Figure 7B). Stroma-high tumors were associated with a significantly shorter OS (HR 2.148, 95%CI 1.322–3.491, *p* = 0.002; Figure 7C). The HR and prognostic performance (AUC = 0.642) of intratumoral histologic stromal percentage in the TCGA COAD cohort were comparable to that of previous reports in colon cancer [12,40]. In the population of the TCGA COAD cohort that was available for TSR scoring (n = 333), the ratio-risk scores demonstrated a prognostic performance similar to that of the histologic stromal percentage (AUC = 0.638; Figure 7D). The prognostic performance marginally improved when both parameters were combined into a single classifier (AUC = 0.671; Figure 7D).

Next, since MSI is associated with increased tumor immunogenicity, we wondered whether the ratio-risk scores were associated with microsatellite status [41]. MSIsensor scores were available for 351 samples. A total of 55 (15.7%) tumor samples demonstrated MSI, as defined by a pre-established cut-off value of ≥4 [24]. On a continuous scale, the ratio-risk scores were not correlated to MSIsensor scores (Rho = 0.082, *p* = 0.127; Figure 7E). However, when applying the defined cut-off values for the MSIsensor scores and the ratio-risk scores, tumors with high ratio-risk scores demonstrated a significantly larger proportion of MSI than low ratio-risk score tumors (21.8 vs. 9.6%, respectively, *p* = 0.003; Figure 7F). As a quality control measure, we observed increased MSIsensor scores in CMS1 tumors, a molecular subtype associated with MSI (Figure 7G).

Subsequently, high ratio-risk score tumors were more often classified as immune (CMS1) and mesenchymal (CMS4) subtypes than low ratio-risk score tumors (19.4 vs. 4.0% and 41.1 vs. 26.3%, respectively, *p* < 0.001; Figure 7H). In accordance with the poor survival of high ratio-risk score tumors and previous literature on CMS survival, CMS1 and CMS4 demonstrated worse survival when compared to CMS2 and CMS3 (*p* = 0.016; Figure 7I) [33]. We then wondered whether combining the CMS classification with the stromal-epithelial ratio-risk scores would improve the prognostic performance in the TCGA-COAD dataset. The CMS classification demonstrated an AUC of 0.487 (Figure 7J). The prognostic performance improved when the CMS classification and the ratio-risk scores were combined into a single classifier (AUC = 0.619) but still underperformed the ratio-risk scores as an individual classifier (AUC = 0.659; Figure 7J).

### 3.7. Validation of Prognostic Performance in an External Dataset

The survival analyses were replicated in an independent dataset to validate the prognostic performance of the stromal-epithelial signature ratio [26]. The median follow-up time was significantly longer in the GSE17538 cohort than in the TCGA COAD cohort (46.5 and 22.3 months, respectively). No additional significant differences in baseline characteristics were observed between the TCGA COAD and GSE17538 cohorts (Supplementary Table S1).

**Figure 8:**
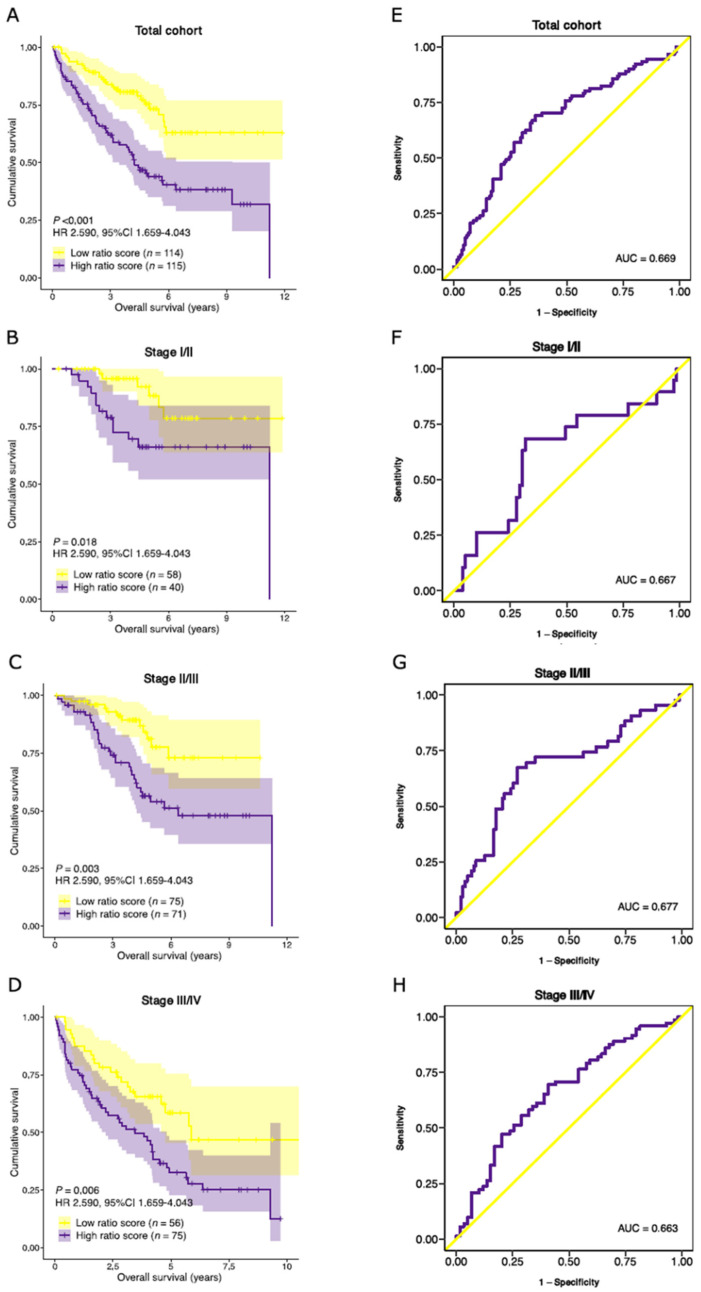
Validation of the prognostic performance of the stromal-epithelial signature ratio in an external cohort. Kaplan-Meier curves of the overall survival time for the stromal-epithelial ratio-risk scores in (**A**) the total cohort (*n* = 229), (**B**) stage I/II (*n* = 98), (**C**) stage II/III (*n* = 146), and (**D**) stage III/IV (*n* = 131) subpopulations. Patients with a high ratio-risk score, categorized by the 50th percentile (median) cut-off, demonstrated a shorter overall survival time. Corresponding area under the receiver operating characteristic curve (AUC) of the stromal-epithelial signature ratio for (**E**) the total cohort, (**F**) stage I/II, (**G**) stage II/III, and (**H**) stage III/IV subpopulations. HR, hazard ratio.

Similar to the TCGA COAD cohort, no significant differences in median ratio-risk scores were observed between different tumor stages (Supplementary Figure S3C,D). Patients with a high ratio-risk score had a lower median OS than those with a low ratio-risk score (46.2 and 48.7 months, respectively). A high ratio-risk score was associated with a significantly shorter OS in the total cohort (HR 2.590, 95%CI 1.659–4.043, *p* < 0.001), as well as in the stage I/II (HR 3.214, 95%CI 1.220–8.467, *p =* 0.018), stage II/III (HR 2.806, 95%CI 1.435–5.485, *p =* 0.003) and stage III/IV (HR 2.030, 95%CI 1.229–3.354, *p =* 0.006) combined subpopulations (Figure 8A–D). The prognostic performance was comparable to the TCGA COAD cohort (Figure 8E–H). Subsequently, multivariate analysis confirmed the independent prognostic performance of the stromal-epithelial signature ratio in OS (HR 2.363, 95%CI 1.485–3.760, *p* < 0.001; Supplementary Table S9).

We then aimed to evaluate the performance of the ratio-risk scores on the DSS. Patients for the GSE17538 cohort were recruited in two separate medical centers [26]. Unfortunately, DSS data were collected in only one of the participating centers and was therefore available for a subset (*n* = 177) of the total cohort. We nevertheless performed survival analyses on the available patients and observed shorter DSS time in patients with a high ratio-risk score (HR 3.045, 95%CI 1.647–5.628, *p* < 0.001) in multivariate analysis (Supplementary Figure S5C & Table S10). Despite the smaller population, the performance of the ratio-risk score was comparable to the TCGA COAD cohort (AUC 0.676 and 0.633, respectively; Supplementary Figure S5D).

## 4. Discussion

The current work introduces a novel approach to gene signature establishment. We report an inventive prognostic risk model of a gene signature ratio established on single-cell transcriptomics data, as opposed to conventional differential expression analyses. The signature ratio, based on genes expressed by stromal-phenotypic and epithelial-phenotypic cells, was associated with colon adenocarcinoma patient survival and proved to be related to our previously discovered histologic TSR [3]. Moreover, the genes included in the signature ratio are constituents of the SLB panel, an experimental stromal-oriented proteomics alternative to conventional liquid biopsy biomarkers. 

Out of the 13 genes included in this study, three genes (TIMP1, PF4 and SERPINA1) were associated with survival and found to be involved in platelet degranulation. In addition, we found enriched expression of genes involved in platelet degranulation in histologic stroma-high tumors, a poor prognostic tumor characteristic. These observations suggest that the secretory function of platelets is a likely determinant of survival in cancer patients, a notion that is supported by previously published reports in various cancer types [42,43,44].

Recently, increased stromal expression of platelet marker CD42b in resectable CRC tumors was associated with poor patient survival [45]. Considering blood-based diagnostics, elevated serum platelet counts and platelet activation cytokine profiles were observed in breast cancer patients with stroma-high tumors in comparison to stroma-low tumors [46]. The authors postulate that stromal-derived factors, such as IL17a and SCF, are likely to be mediators of platelet production and activation. Moreover, PF4, a constituent of the SLB panel, was shown to promote the production of tumor-educated platelets in lung cancer, a platelet subpopulation that stimulates tumor progression and is currently appreciated as a potential analyte in liquid biopsy [47,48]. Despite these observations, the mechanistic association between platelet activation and intratumoral stromal content in colon cancer remains to be elucidated and should be the subject of future work.

Interestingly, TIMP1 and SERPINA1 were identified as hub genes during network analysis, implying that TIMP1 and SERPINA1 share multiple functional interactions with the other genes included in this study and are therefore likely to host a regulatory role in stromal processes. Moreover, both of these proteins are protease inhibitors, and as such may play key roles in regulating proteolytic mechanisms. Noteworthy, we observed an unexpected decrease in prognostic performance of the stromal-epithelial signature ratio when the non-significant genes were excluded from the original composition. This strongly suggests that the overall performance of a gene signature is greater than the sum of its parts. Commonly applied methods for prognostic gene signature construction rely on supervised top-down approaches, where genes are selected for inclusion based on their prognostic performance [49]. Our results provide support for a bottom-up approach, where biological hypotheses drive robust gene signature construction, even when its constituents are not individually associated with survival. We believe that the workflow presented here may inspire future (gene) signature development based on biological frameworks. 

The significance of assessing the TME for cancer diagnostics is highlighted by the prognostic success of the TSR [3,12,50]. In addition, in an explorative study, we recently demonstrated that combined assessment of intratumoral stromal content and tumor immune cell infiltrate may provide for a clinically feasible biomarker for therapy response prediction [51]. In the current study, we have demonstrated that histologic high stromal content is accompanied by increased gene expression of stroma-associated pathways in comparison to tumors with low stromal content. The gene signature ratio described in this study was found to be related to the TSR and demonstrated a remarkably similar prognostic performance. The prognostic performance only marginally improved when the two parameters were combined into a single classifier, suggesting that the two parameters are likely to capture similar populations at risk. These findings provide further molecular evidence for the prognostic power of the tumor stroma in clinical practice. 

In addition to patient prognosis, high gene signature ratio-risk scores were associated with an increased proportion of MSI in comparison to low ratio-risk scores. MSI occurs in roughly 15–20% of the colon carcinomas and has implications for immune checkpoint inhibitor (ICI) therapy [33,52]. Indeed, in a recent feasibility study of neoadjuvant ICI therapy in early-stage colon cancer, a pathologic response was observed in 20/20 MSI tumors and 4/15 MSS tumors [53]. Given the increased proportion of MSI in the high ratio-risk score group, the signature ratio might be predictive of ICI therapy response in colon cancer and should be the subject of future studies in ICI therapy-treated patient cohorts.

Predictably, the TSR and the established CMS classification for CRC did not demonstrate significant overlap. The heterogeneity of tumor immune cell infiltration in the stromal compartment, recently demonstrated by us and others, is not adequately captured by the CMS classification system [51,54,55]. As an illustration, CMS2 tumors are characterized by increased wnt-pathway signaling and an immune-depleted TME [33]. We observed increased wnt-pathway gene expression in stroma-high tumors in comparison to stroma-low tumors. Yet, the proportion of CMS2 tumors did not significantly differ between stroma-high and stroma-low tumors. This is likely explainable by the vast heterogeneity of immune cell infiltration in histologic stroma-high and stroma-low tumors [51]. The TSR fails to account for tumor immune cell infiltrate. Consequently, the TSR and the CMS classification demonstrate limited overlap and are therefore likely to capture different populations at risk. Current and future tumor classification systems should be tailored toward unmet clinical needs in oncology. Given the current absence of TME involvement in cancer staging and diagnostics, we plead for the implementation of the TSR in routine pathology. The TSR is currently the subject of prospective validation in colon carcinoma in the international UNITED study [36].

Despite the promising results of the TSR, its evaluation requires access to solid tumor tissue. In recent years, liquid biopsy, characterizing disease status by the sampling of blood, has rapidly gained momentum in cancer biomarker research [56]. However, initial efforts have emphasized CTCs and ctDNA, without regard to the TME [57]. Recently, the SLB panel, a protein panel believed to capture the proteomic blueprint of a cancer-associated systemic response, was developed for the evaluation of TME characteristics [20]. A selection of the proteins from this panel are of high abundance in plasma (i.e., C3 and SERPINA1), while others are not (i.e., PF4 and ECM1), and so reflect the contributions of localized changes due to tumor presence and anti-tumor immunity. Interestingly, we observed increased gene expression of the SLB panel constituents in stroma-high tumors in comparison to stroma-low tumors. If intratumoral stromal content is reflected in the plasma proteome, increased plasma abundance of SLB panel proteins may indicate the presence of a stroma-rich tumor and therefore provide valuable prognostic information. Future work should focus on the association between SLB protein abundance in plasma and histologic intratumoral stroma content. 

The current report functionally characterizes a selection of the genes comprising the protein panel and provides a first theoretical framework for its effectiveness in liquid biopsy. By combining bioinformatics tools with a novel liquid biopsy concept, we were able to identify a prognostic biomarker and apply it to colon cancer. The composition of the signatures described here could potentially serve as an indicator for tumor-stroma content when applied in liquid biopsy. Ultimately, the TME protein blueprint, as captured by the SLB panel, may provide a more refined stratification of the tumor and patient prognosis, and offer new insights into therapeutic strategies that might beneficially modulate the TME. 

We acknowledge the limitations of our study. Although the prognostic performance of the signature ratio remained statistically significant in subpopulation analyses, for better interpretable results, further analyses in larger subpopulation cohorts are warranted. In addition to limited sample size, only a limited selection of clinical covariates was available for multivariate analyses, therefore we were unable to account for potential confounders, other than those presented here. In particular, treatment data from both cohorts included in this study were incomplete or missing. Although the baseline characteristics of the compared populations were not statistically significantly different, no definitive conclusions can be drawn concerning the treatment-independent prognostic performance of the gene signature ratio. This should therefore be investigated in future studies. Next, we used the median ratio-risk score as a cut-off for the discrimination into high-risk and low-risk groups. The prognostic performance is likely to improve when applying discriminant analysis to determine the cut-off value. Lastly, as a general limitation in cancer molecular research, bulk RNA sequencing may fail to capture the vast tumor heterogeneity and may therefore have limited interpretability. Nevertheless, we aimed to illustrate a novel concept of stromal-expressed versus epithelial-expressed genes as a predictor of survival. 

## 5. Conclusions

The current work describes a stromal-epithelial gene signature ratio established on single-cell transcriptomics data and constituents of the SLB proteomics panel. The gene signature ratio was found to be a predictor of survival in colon adenocarcinoma and was subsequently validated with success in an external cohort. Moreover, the signature ratio and the expression of SLB genes were associated with the histologic TSR, providing further molecular background for its prognostic accomplishment in cancer. Lastly, future work should focus on the plasma protein levels of the genes described here, as they may reflect histologic intratumoral stromal content and hence provide valuable prognostic information in liquid biopsy. Ultimately, we believe that the results described above contribute to the ever-increasing appreciation of the TME in cancer. 

## Figures and Tables

**Table 1:** Functional annotations of the 13 stroma-derived genes from the *Stromal Liquid Biopsy* panel.

Gene	HGNC Symbol	Ensemble ID	Pathway Based on Literature
Complement component 3	C3	ENSG00000125730	Complement cascade
Complement factor B	CFB	ENSG00000243649	Complement cascade
Complement factor properdin	CFP	ENSG00000126759	Complement cascade
Complement component 4 binding protein alpha	C4BPA	ENSG00000123838	Complement cascade
Platelet factor 4	PF4	ENSG00000163737	Coagulation
Pro-platelet basic protein	PPBP	ENSG00000163736	Coagulation
Thrombospondin 1	THBS1	ENSG00000137801	Coagulation
TIMP metallopeptidase inhibitor 1	TIMP1	ENSG00000102265	Coagulation
Chromogranin A	CHGA	ENSG00000100604	Acute-phase inflammation
Extracellular matrix protein 1	ECM1	ENSG00000143369	Acute-phase inflammation
Serum amyloid A2	SAA2	ENSG00000134339	Acute-phase inflammation
Serpin Family A Member 1	SERPINA1	ENSG00000197249	Multiple
Serpin family D member 1	SERPIND1	ENSG00000099937	Multiple

HGNC, HUGO Gene Nomenclature Committee.

## Data Availability

RNA-seq data used in this study is publicly available on The Cancer Genome Atlas (TCGA) COAD project (https://portal.gdc.cancer.gov/). Patient/sample identifiers used in this study are provided in the supplementary data. The complete single-cell RNA-seq dataset used in this study and is publicly available for exploration at http://crcleukocyte.cancer-pku.cn/. Processed filtered single-cell RNA-seq data are provided in the supplementary data. Novel computed scores (i.e., ratio-risk scores, TSR scores, and gene set enrichment scores) are provided in the supplementary data. Any additional data are available from the corresponding author upon reasonable request.

## Supplementary Materials

The following are available online at https://www.mdpi.com/article/10.3390/cancers14010163/s1. Figure S1: Equation for the stromal-epithelial ratio risk score. Figure S2: PPI networks for the SLB gene set. Figure S3: Distribution of stromal-epithelial ratio-risk scores in the discovery and validation cohorts. Figure S4: AUCs of the stromal-epithelial ratio-risk score in the discovery and validation cohorts. Figure S5: DSS in the discovery and validation cohorts. Table S1: Baseline characteristics of the discovery and validation cohorts. Table S2: Regression coefficients as computed for the discovery and validation cohorts. Table S3: Functional enrichment analysis of the SLB gene set. Table S4: Network statistics and hub genes. Table S5: Baseline characteristics of the stroma-high and stroma-low populations. Table S6: Cox proportional hazards model of the OS in the discovery cohort. Table S7: Baseline characteristics of the high ratio-risk score and low ratio-risk score populations. Table S8: Cox proportional hazards model of the DSS in the discovery cohort. Table S9: Cox proportional hazards model of the OS in the validation cohort. Table S10: Cox proportional hazards model of the DSS in the validation cohort. Data S1: Patient identifiers and novel computed scores. Data S2: Signature construction.
