# Supplementary material for: The Stroma Liquid Biopsy Panel Contains a Stromal-Epithelial Gene Signature Ratio That Is Associated with the Histologic Tumor-Stroma Ratio and Predicts Survival in Colon Cancer
Source: Cancers (Basel). 2021 Dec 29;14(1):163. doi: 10.3390/cancers14010163 (PMC8750571; doi:10.3390/cancers14010163)
Supplement: Supplementary file 1 [file cancers-14-00163-s001.zip › Supplementary_Figures_Tables.pdf]

Supplement to:

The Stroma Liquid Biopsy panel contains a stromal-epithelial gene signature ratio that is associated with the histologic tumor-stroma ratio and predicts survival in colon cancer

C.J. Ravensbergen[1], M. Kuruc[2], M. Polack[1], A.S.L.P. Crobach[3], H. Putter[4], A.J. Gelderblom[5], D. Roy[6], R.A.E.M. Tollenaar[1†] & W.E. Mesker[1†*]

[1]Department of Surgery, Leiden University Medical Center, Leiden, the Netherlands
[2]Biotech Support Group LLC, Monmouth Junction, USA
[3]Department of Pathology, Leiden University Medical Center, Leiden, the Netherlands
[4]Department of Medical Statistics, Leiden University Medical Center, Leiden, the Netherlands
[5]Department of Medical Oncology, Leiden University Medical Center, Leiden, the Netherlands
[6]Hospital medicine, Nathan Littauer Hospital, Gloversville, USA

[†]Contributed equally
[*]Correspondence to: Dr Wilma E. Mesker, Department of Surgery, Leiden University Medical Center, Albinusdreef 2, Leiden, 2300RC, the Netherlands. Tel: +31715262987. Mail: W.E.Mesker@lumc.nl

$$\left[\frac{\text{Stromal risk score} = \sum_{i=1}^{n} ß_i \ * \ E_i}{\text{Epithelial risk score} = \sum_{j=1}^{n} ß_j \ * \ E_j} \right]^2$$

Supplementary Figure S1. The equation for the stromal-epithelial ratio-risk score. In the above equation, n is the number of genes in the Stromal signature (i) or the Epithelial signature (j); ß represents the regression coefficient and E represents the absolute expression value of the gene.

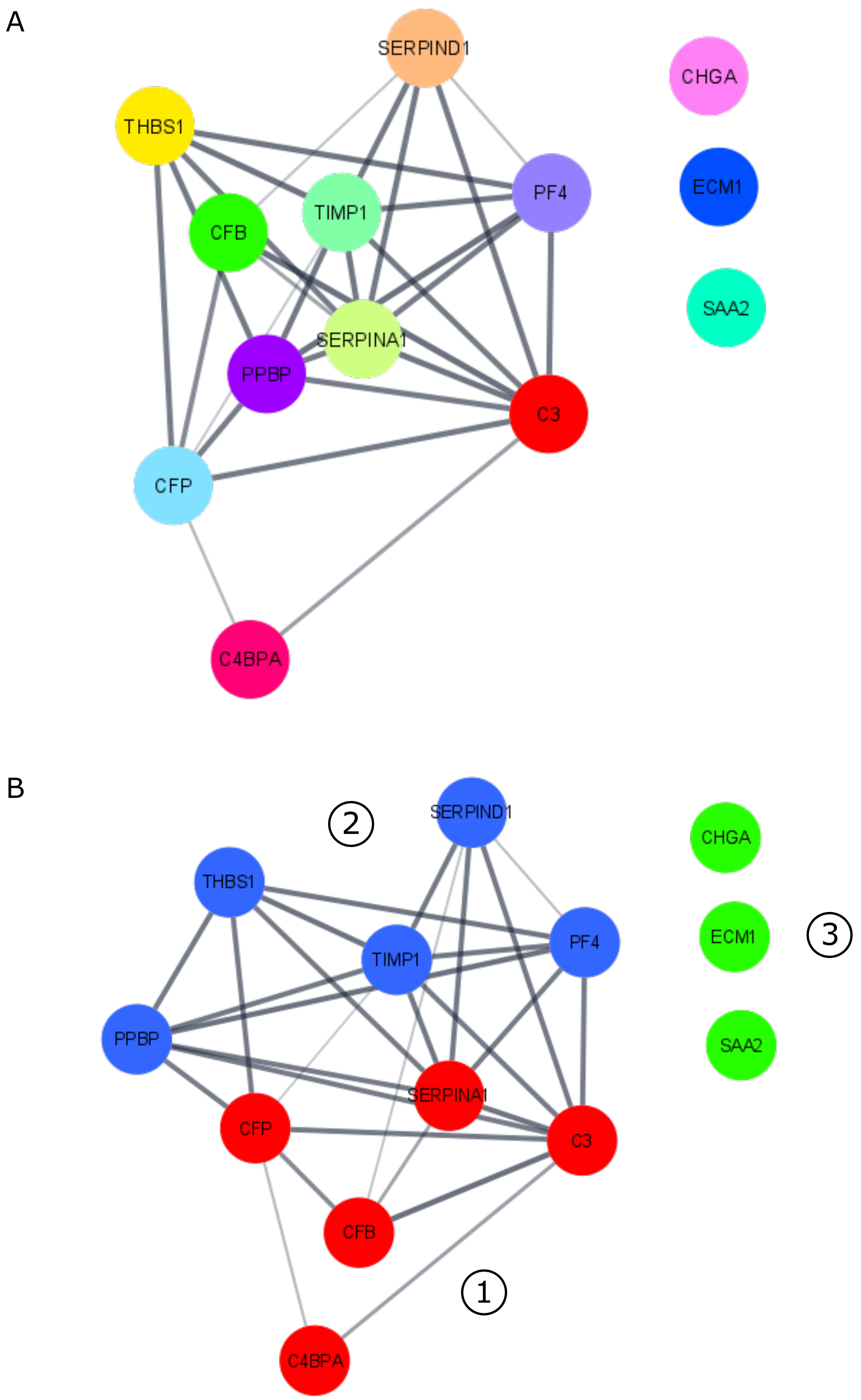

Supplementary Figure S2. Protein-protein interaction (PPI) networks of the Stromal Liquid Biopsy (SLB) gene set. **(A)** PPI displaying functional interactions between the SLB genes. Thickness of the edges represents the strength of scientific evidence. **(B)** The PPI network stratified into three clusters based on K-means clustering. The clusters demonstrate similar content as the functional annotations described in *Table 1*.

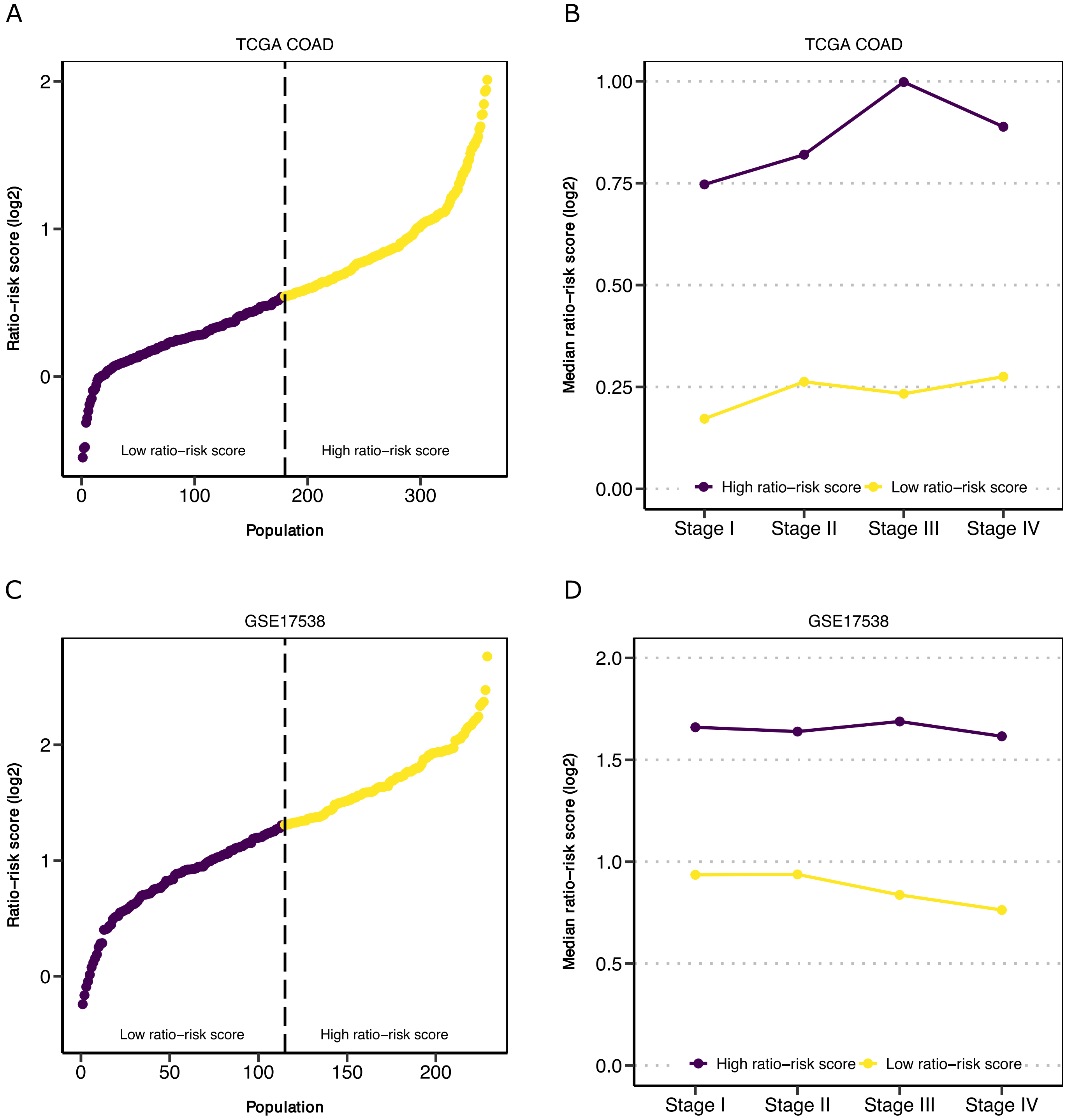

Supplementary Figure S3. Distribution of stromal-epithelial ratio-risk scores. The 50th percentile (median) was used to categorize patients into high and low ratio-risk scores in **(A)** the TCGA Pan-Cancer COAD discovery cohort and **(C)** the GSE17538 validation cohort. Median ratio-risk score stratified by stage is displayed for **(B)** the TCGA Pan-Cancer COAD cohort and **(D)** the GSE17538 cohort.

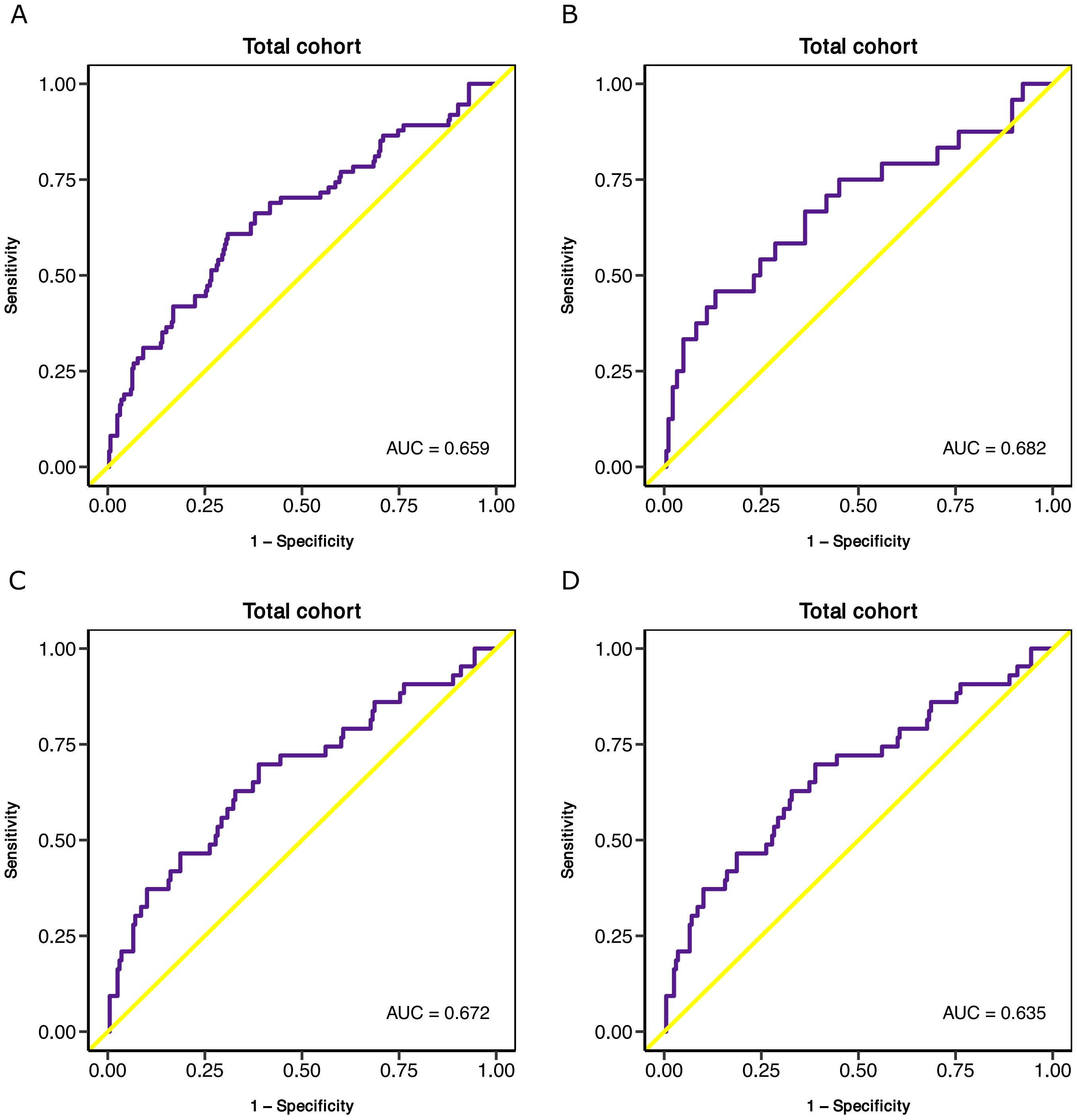

Supplementary Figure S4. Area under the receiver operating characteristic curve (AUC) of the stromal-epithelial ratio-risk score for **(A)** the TCGA Pan-Cancer COAD total cohort, **(B)** stage I/II, **(C)** stage II/III and, **(D)** stage III/IV subpopulations.

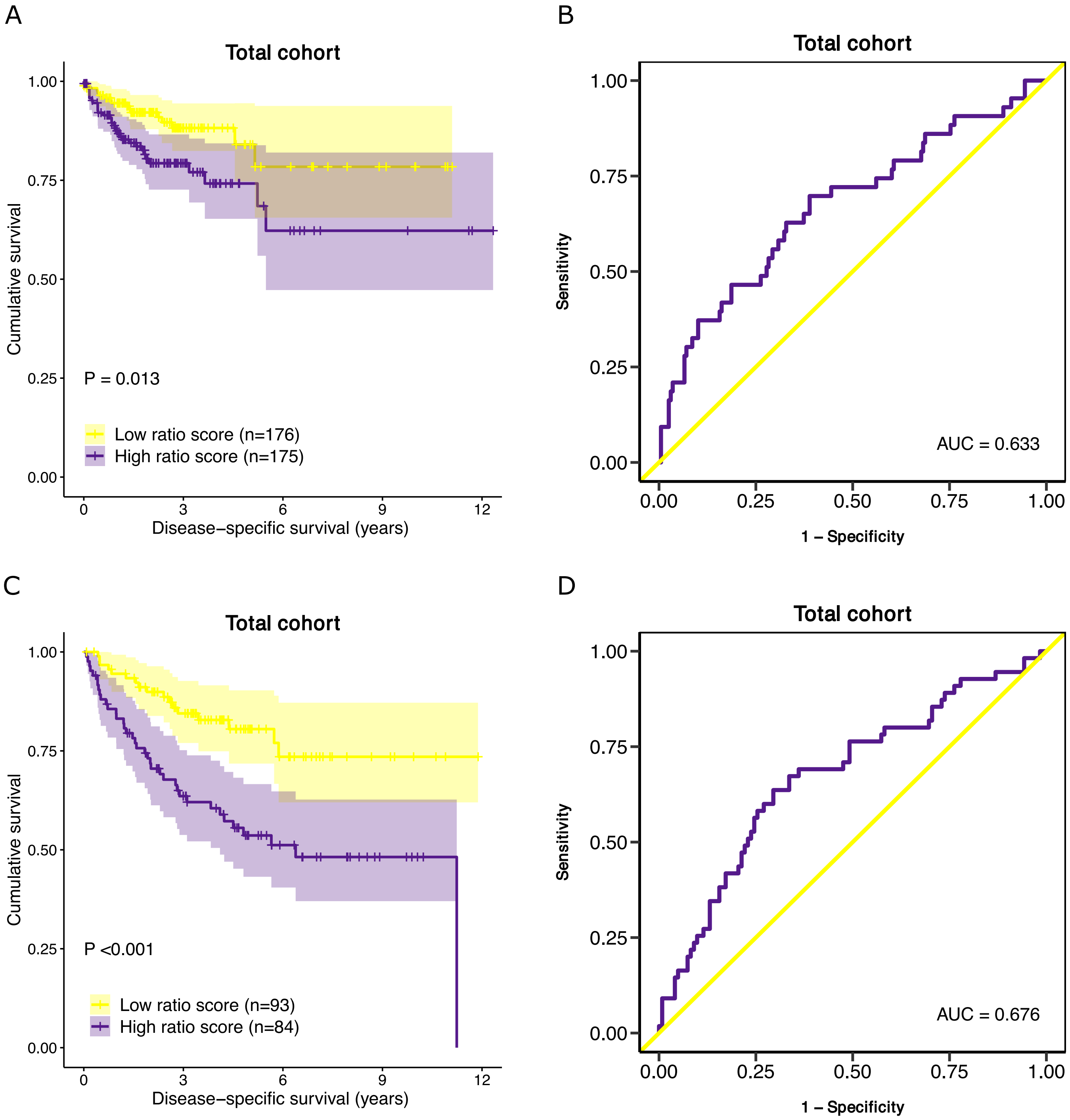

Supplementary Figure S5. Disease-specific survival (DSS) in the TCGA Pan-Cancer COAD discovery cohort and the GSE17538 validation cohorts. **(A)** Kaplan-Meier curve of the DSS for the total TCGA COAD cohort (n = 351) and **(B)** the corresponding area under the receiver operating characteristic curve (AUC). **(C)** Kaplan-Meier curve of the DSS for the available data of the GSE17538 cohort (n = 177) and **(D)** the corresponding AUC.

Supplementary Table S1. Baseline characteristics of the TCGA COAD discovery cohort and the GSE17538 validation cohort. NA, not applicable.

	**TCGA COAD (n = 359)**	**GSE17538 (n = 229)**	**P-value**
Age (%)			
<60	98 (27.3)	79 (34.5)	0.051†
60-75	159 (44.3)	100 (43.7)	
>75	102 (28.4)	50 (21.8)	
Gender (%)			
Female	168 (46.8)	109 (47.6)	0.894‡
Male	191 (53.2)	120 (52.4)	
Histologic subtype (%)			
Adenocarcinoma	359 (100)	229 (100)	NA
Other	0 (0)	0 (0)	
TNM (%)			
I	64 (17.8)	28 (12.2)	0.803†
II	142 (39.6)	70 (30.6)	
III	99 (27.6)	76 (33.2)	
IV	54 (15.0)	55 (24.0)	
Neoadjuvant therapy (%)			
Yes	0 (0)	0 (0)	NA
No	359 (100)	229 (100)	
Radiation therapy (%)			
Yes	6 (1.7)	0 (0)	NA
No	300 (83.6)	0 (0)	
Missing	53 (14.7)	229 (100)	
Systemic therapy (%)			
Chemotherapy	89 (24.8)	0 (0)	NA
Chemo + molecular therapy	31 (8.6)	0 (0)	
Missing	239 (66.6)	229 (100)	
Median follow-up time (months)	22.3	46.5	<0.001§

†Chi-squared test
‡Fisher's exact test
§Mann-Whitney U test

Supplementary Table S2. Regression coefficients as computed for the TCGA Pan-Cancer COAD cohort and GEO17538 validation cohort.

	Stromal signature			Epithelial signature	
Gene	TCGA COAD	GEO17538	Gene	TCGA COAD	GEO17538
C3	0.058	-0.178	C4BPA	0.051	0.002
CFP	-0.155	-0.753	CFB	-0.096	0.002
ECM1	-0.165	-0.067	CHGA	0.035	0.102
THBS1	0.090	0.379	PF4	-0.178	-0.613
TIMP1	0.416	0.658	PPBP	0.055	0.12
			SAA2	0.116	-0.074
			SERPINA1	-0.192	0.128
			SERPIND1	-0.115	-0.039

Supplementary Table S3. Functional enrichment analysis demonstrating the top five tumor-associated gene ontology (GO) biological processes associated with the Stroma Liquid Biopsy panel gene set. FDR, false discovery rate.

GO Biological Process	Fold enrichment	Involved genes	FDR
GO:0006957 Complement activation, alternative pathway	>100	C3, CFB, CFP	1.93E-04
GO:0002576 Platelet degranulation	74.26	ECM1, PF4, PPBP, SERPINA1, THBS1, TIMP1	4.40E-07
GO:0030449 Regulation of complement activation	54.16	C3, C4BPA, CFB, CFP	6.44E-04
GO:0097529 Myeloid leukocyte migration	38.02	CHGA, PF4, PPBP	1.87E-02
GO:0045766 Positive regulation of angiogenesis	30.47	C3, ECM1, THBS1	3.26E-02

Supplementary Table S4. Network statistics of hub genes displaying node degree count, centrality statistics and assigned K-means cluster.

Gene/Protein	Degree	Closeness centrality	Betweenness centrality	Assigned to cluster
C3	8	0.90	0.18	1
TIMP1	7	0.82	0.04	2
SERPINA1	7	0.82	0.06	1
PPBP	6	0.75	0.02	2
PF4	6	0.75	0.02	2
CFP	6	0.75	0.11	1
THBS1	5	0.69	0.01	2
SERPIND1	5	0.69	0.02	2
CFB	4	0.64	0.01	1
C4BPA	2	0.56	0	1
CHGA	0	0	0	3
ECM1	0	0	0	3
SAA2	0	0	0	3

Supplementary Table S5. Baseline characteristics of the stroma-high and stroma-low populations from the TCGA Pan-Cancer COAD cohort, as scored by the tumor-stroma ratio.

	**Stroma-high (n = 131)**	**Stroma-low (n = 202)**	**P-value**
Age (%)			
<60	43 (32.8)	48 (23.8)	0.617†
60-75	52 (39.7)	96 (47.5)	
>75	36 (27.5)	58 (28.7)	
Gender (%)			
Female	69 (52.7)	96 (47.5)	0.862‡
Male	62 (47.3)	106 (52.5)	
pTNM (%)			
I	21 (16.0)	39 (19.3)	0.896†
II	54 (41.2)	76 (37.6)	
III	37 (28.2)	58 (28.7)	
IV	19 (14.5)	29 (14.4)	
pT-stage (%)			
T1	2 (1.5)	7 (3.5)	0.345†
T2	21 (16.0)	39 (19.3)	
T3	91 (69.5)	138 (68.3)	
T4	17 (13.0)	18 (8.9)	
pN-stage (%)			
N0	76 (58.0)	120 (59.4)	0.852†
N1	38 (29.0)	43 (21.3)	
N2	17 (13.0)	39 (19.3)	

†Chi-squared test
‡Fisher's exact test

Supplementary Table S6. Cox proportional hazards model of univariate and multivariate analyses for the overall survival of colon adenocarcinoma patients from the TCGA Pan-Cancer COAD cohort. HR, hazard ratio; CI, confidence interval.

	**Overall survival**						
		**Univariate analysis**			**Multivariate analysis**		
	n	HR	95% CI	P-value	HR	95% CI	P-value
Age							
<60	98			0.031			0.007
60-75	159	1.479	0.785-2.788		2.059	1.057-4.011	
>75	102	2.287	1.207-4.334		2.977	1.503-5.897	
Gender							
Female	168			0.294			0.995
Male	191	1.282	0.806-2.041		1.002	0.617-1.627	
pTNM							
I	64			<0.001			
II	142	1.324	0.614-5.309				
III	99	2.433	1.143-9.601				
IV	54	7.399	3.420-28.03				
pT-stage							
T1	9			<0.001			0.010
T2	64	0.593	0.061-5.744		0.353	0.036-3.485	
T3	246	2.226	0.307-16.12		1.014	0.135-7.636	
T4	40	6.755	0.888-51.39		2.281	0.284-18.35	
pN-stage							
N0	214			<0.001			<0.001
N1	84	1.959	1.112-3.451		1.931	1.061-3.512	
N2	61	3.908	2.275-6.715		3.638	2.027-6.532	
Ratio-risk							
score	179			<0.001			<0.001
Low	180	2.581	1.567-4.251		2.586	1.561-4.286	
High							

Supplementary Table S7. Baseline characteristics of the high ratio-risk score and the low ratio-risk score tumor populations from the TCGA Pan-Cancer COAD cohort.

	**High ratio-risk score (n = 180)**	**Low ratio-risk score (n = 179)**	**P-value**
Age (%)			
<60	47 (26.1)	51 (28.5)	0.656†
60-75	78 (43.3)	81 (45.3)	
>75	55 (30.6)	47 (26.2)	
Gender (%)			
Female	90 (50.0)	78 (43.6)	0.265‡
Male	90 (50.0)	101 (56.4)	
pTNM (%)			
I	29 (16.1)	35 (19.5)	0.442†
II	72 (40.0)	70 (39.1)	
III	47 (26.1)	52 (29.1)	
IV	32 (17.8)	22 (12.3)	
pT-stage (%)			
T1	4 (2.2)	5 (2.8)	0.227†
T2	29 (16.1)	35 (19.6)	
T3	121 (67.2)	125 (69.8)	
T4	26 (14.5)	14 (7.8)	
pN-stage (%)			
N0	104 (57.8)	110 (61.5)	0.778†
N1	44 (24.4)	40 (22.3)	
N2	32 (17.8)	29 (16.2)	

†Chi-squared test
‡Fisher's exact test

Supplementary Table S8. Cox proportional hazards model of univariate and multivariate analyses for the disease-specific survival of colon adenocarcinoma patients from the TCGA Pan-Cancer COAD cohort. HR, hazard ratio; CI, confidence interval.

		**Disease-specific survival**					
		**Univariate analysis**			**Multivariate analysis**		
	n	HR	95% CI	P-value	HR	95% CI	P-value
Age							
<60	98			0.508			0.275
60-75	153	1.036	0.513-2.091		1.444	0.696-2.997	
>75	100	1.445	0.701-2.980		1.879	0.870-4.058	
Gender							
Female	167			0.730			0.806
Male	184	1.103	0.631-1.928		0.929	0.518-1.668	
pTNM							
I	64			<0.001			
II	137	1.536	0.319-7.406				
III	96	4.491	1.020-19.77				
IV	54	19.89	4.723-83.75				
pT-stage							
T1	9			<0.001			0.012
T2	64	0.175	0.011-2.818		0.122	0.007-2.001	
T3	238	1.473	0.201-10.80		0.642	0.082-5.027	
T4	40	5.239	0.681-40.32		1.589	0.187-13.48	
pN-stage							
N0	209			<0.001			<0.001
N1	83	3.263	1.584-6.721		2.725	1.275-5.825	
N2	59	6.366	3.163-12.82		5.218	2.460-11.07	
Ratio-risk							
score	176			0.013			0.014
Low	175	2.099	1.169-3.770		2.107	1.162-3.817	
High							

Supplementary Table S9. Cox proportional hazards model of univariate and multivariate analyses for the overall survival of colon adenocarcinoma patients from the GSE17538 cohort. HR, hazard ratio; CI, confidence interval.

		**Overall survival**					
		**Univariate analysis**			**Multivariate analysis**		
	n	HR	95% CI	P-value	HR	95% CI	P-value
Age							
<60	79			0.012			<0.001
60-75	100	0.822	0.505-1.338		1.560	0.926-2.626	
>75	50	1.772	1.047-3.000		3.417	1.977-5.906	
Gender							
Female	109			0.793			
Male	120	0.946	0.625-1.431				
pTNM							
I	28			<0.001			<0.001
II	70	1.761	0.582-5.330		1.798	0.592-5.458	
III	76	3.310	1.142-9.595		3.509	1.203-10.23	
IV	55	12.91	4.546-36.67		14.16	4.915-40-78	
Ratio-risk							
score	114			<0.001			<0.001
Low	115	2.590	1.659-4.043		2.363	1.485-3.760	
High							

Supplementary Table S10. Cox proportional hazards model of univariate and multivariate analyses for the disease-specific survival of colon adenocarcinoma patients from the GSE17538 cohort. HR, hazard ratio; CI, confidence interval.

	Disease-specific survival						
		Univariate analysis			Multivariate analysis		
	n	HR	95% CI	P-value	HR	95% CI	P-value
Age							
<60	55			0.387			0.168
60-75	80	0.672	0.369-1.224		1.192	0.630-2.254	
>75	42	0.949	0.464-1.940		2.033	0.959-4.311	
Gender							
Female	81			0.534			0.725
Male	96	1.187	0.692-2.035		0.901	1.647-5.628	
pTNM							
I	24			<0.001			<0.001
II	57	4.734	0.595-37.65		3.826	0.476-30.73	
III	57	9.863	1.271-76.56		7.650	0.992-59.02	
IV	39	49.79	6.516-380.5		49.44	6.486-376.80	
Ratio-risk							
score	93			<0.001			<0.001
Low	84	2.729	1.540-4.838		3.045	1.647-5.628	
High							
